# Welding Joints in High Entropy Alloys: A Short-Review on Recent Trends

**DOI:** 10.3390/ma13061411

**Published:** 2020-03-20

**Authors:** Fabio C. Garcia Filho, Sergio N. Monteiro

**Affiliations:** Department of Materials Science, Military Institute of Engineering—IME, Praça General Tibúrcio 80, 22290-270 Rio de Janeiro/RJ, Brazil; snevesmonteiro@gmail.com

**Keywords:** high entropy alloys, welding techniques, welding zone microstructure, welding joint properties

## Abstract

High entropy alloys (HEAs) emerged in the beginning of XXI century as novel materials to “keep-an-eye-on”. In fact, nowadays, 16 years after they were first mentioned, a lot of research has been done regarding the properties, microstructure, and production techniques for the HEAs. Moreover, outstanding properties and possibilities have been reported for such alloys. However, a way of jointing these materials should be considered in order to make such materials suitable for engineering applications. Welding is one of the most common ways of jointing materials for engineering applications. Nevertheless, few studies concerns on efforts of welding HEAs. Therefore, it is mandatory to increase the investigation regarding the weldability of HEAs. This work aims to present a short review about what have been done in recent years, and what are the most common welding techniques that are used for HEAs. It also explores what are the measured properties of welded HEAs as well as what are the main challenges that researchers have been facing. Finally, it gives a future perspective for this research field.

## 1. Introduction

High entropy alloys (HEAs) emerged in the beginning of this century as promising materials for engineering applications. Their unique compositions, microstructure, and properties are the most attractive characteristics and an incredible number of papers have been published regarding this subject. According to the Scopus database, publications include more than 7,000 research papers, 300 review articles, and eight books over the 16 years, since these alloys were firstly reported by Cantor’s [1] and Yeh’s research groups [2,3,4,5,6]. One should be wondering what make these alloys so special. In fact, the multi-main element in contrast to the traditional just one or two main elements that mankind have been using in the development of metallic alloys over the centuries leads to interesting discoveries. Multi-main elements would be responsible for increasing the configurational entropy of the alloy and, therefore, favoring a single phase material instead of a microstructure with several phases [6]. This incredible microstructure led to the remarkable mechanical and functional properties reported for these alloys [7,8,9,10,11,12,13]. Gludovatz et al. [7] studied the fracture resistance of HEAs. The authors reported that these HEAs were able to combine high fracture toughness, above 10^2^ MPa.m^1/2^, with high yield strength, above 1.1 GPa. Moreover, these HEAs exhibit the highest relation between strength and ductility among all materials when compared to other class of materials as stainless steels, low alloy steel, nickel-based super alloys, metallic glasses, polymers, and ceramics, even at cryogenic temperature. This is a notable characteristic of these alloys that encourages their use in engineering applications. Nevertheless, it is critical to understand the behavior of these alloys during the processing and fabrication in order to achieve practical use. 

Welding is a fabrication technology that is used in a variety of industries. Moreover, it is certainly the most used jointing techniques for metallic materials. Welding techniques could be divided into solid and liquid state processes. Liquid-state welding relies on the fusion of the metal to make the weld. Techniques that are based on gas oxyacetylene, shield metal arc (SMAW), gas-tungsten arc (GTAW), gas-metal arc (GMAW), electroslag (ESW), and others, as well as high-energy beam such as electron beam (EBW) and laser beam (LBW) welding are the most commonly used liquid-state ones [14]. Solid-state welding is defined as a joining process without any liquid/vapor phase formation and with the use of pressure. Friction (FW) and friction stir (FSW) welding are the some notable techniques in such class [15,16,17,18]. It is obvious that each welding technique presents significant differences in terms of joint preparation and sample thickness, as well as speed and energy input into the welding joint. For instance, the heat density of a GMAW technique is around 10^5^ W/cm^2^, while, for the EBW, this density can reach 10^8^ W/cm^2^ [19]. Such differences will directly impact the quality and properties of the welded joint [20,21].

Few works actually combined both subjects despite the aforementioned importance of the HEAs and the commonly used welding techniques for jointing metallic materials. When comparing the number of publications regarding HEAs properties or characterization and those that somehow discussed the welding in these alloys, less than 0.5% indeed concerned it. The behavior of a novel alloy during welding is considered to be a key technological issue. HEAs are no exception, and the behavior of the alloy when exposed to a welding thermal cycle needs to be explored and understood. The investigation of the alloy can be welded or joined without degradation, whether that is detrimental to the weldment microstructure or properties (during/after welding) and for the duration of intended service, is mandatory for its potential use as an engineering material for structural applications [22]. In this context, this short-review objective is to cover what has been reported on HEAs welded so far, including the influence of the welding techniques and the parameters in the material’s properties and microstructure, as well as to critically assess the achievements reported and outlook what of is yet to come in future years. 

## 2. Welding Techniques in HEAs 

**Figure 1:**
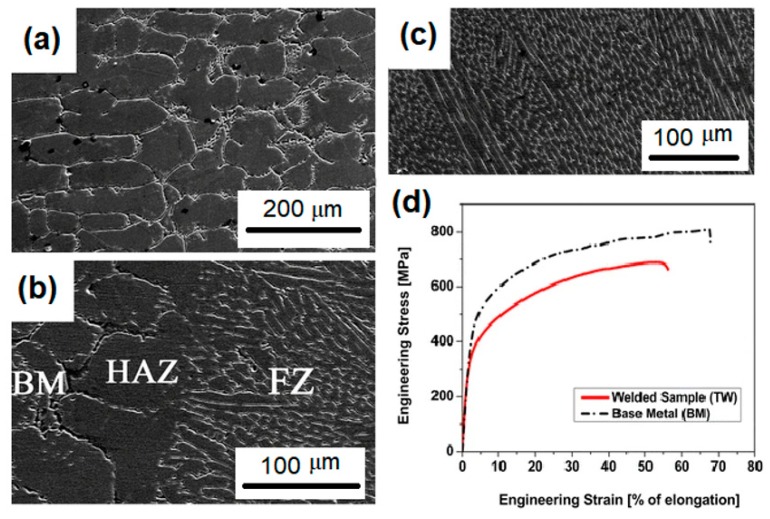
Gas-tungsten arc (GTAW) technique to weld an Al_0.5_CoCrFeNi alloy (**a**) base metal zone (BM) microstructure, (**b**) BM+HAZ+FZ region, (**c**) FZ microstructure, and (**d**) comparison of the mechanical strength of the BM and welded sample. Adapted from [23].

Gas tungsten arc welding (GTAW) is a well-known technique among the arc welding techniques for jointing metallic materials, especially high alloy materials [14]. In this process, a non-consumable tungsten electrode is used to produce the welded joint by melting the base metal. In addition, the weld area is protected from atmospheric contamination by an inert shielding gas, such as argon or helium. In one of the first papers regarding the welding of HEAs, Sokkalingam et al. [23] used the GTAW technique to weld an Al_0.5_CoCrFeNi alloy. The parameters of 40 A for current, 12 V for voltage, and 80 mm min^-1^ for weld velocity were chosen to joint 2.5 mm HEA plates. The main achievement of this work was a remarkable refinement of the grains from 60 μm in the base metal to a range of 8–12 μm grain size in the fusion zone. Nevertheless, a reduction of approximately 6.4 and 16.5 % were reported for strength and ductility, respectively in comparison with the base metal. Figure 1 displays the microstructure of the welded zones as well as the measured tensile properties of that work. Similarly, Wu et al [24] welded a CoCrFeMnNi alloy while using GTAW. Sheets with 1.6 mm of thickness were butt-welded using a voltage of 8.4 V, current of 75 A, and velocity of 25.4 mm min^−1^ as the welding parameters. No cracks or significant microsegregation were produced. 

**Figure 2:**
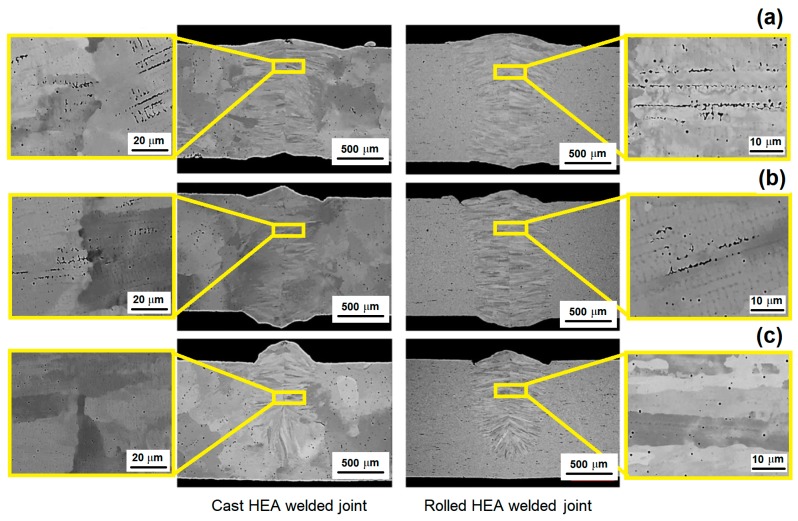
Comparison of the welding microstructure of the CoCrFeMnNi alloy as cast (left) and rolled (right). Samples welded by LBW in different velocities (**a**) 6 m min^−1^, (**b**) 8 m min^−1^, and (**c**) 10 m min^−1^. Adapted from [26].

Recently, high-energy beam welding were reported in several publications regarding high entropy alloys weld [25,26,27,28]. The high concentrated heat from laser or electron beam create a narrow heat affected zone in the material and allow for high welding rates to be achieved in comparison to arc welding techniques. Chen et al. [25] were able to successfully use high-power solid-state laser to weld CoCrFeMnNi high entropy alloy plates. The laser power was 2 kW, defocusing amount of +2 mm, laser spot moving rate of 1 m min^−1^, and shielding argon gas flow rate of 30–40 L min.^-1^ were the parameters used in YLS10000 fiber laser (American IPG Company, Oxford, MA, USA to produce the welded joint. When considering the same composition HEA Cantor alloy processed in two different conditions (cast and rolled), Nam et al. [26] used laser beam welding to study the impact of different welding velocities in their microstructures. The butt welding specimens dimensions were 100 × 20 × 1.5 mm^3^ and Nd:YAG laser power of 3.5 kW, beam diameter of 300 μm, and focal legth of 304 mm, with no shielding gas was used for welding. The welding velocity was in the range of 6–10 m min^−1^. Figure 2 exhibits the result of such investigation. The authors reported the good weldability of the CoCrFeMnNi alloy with no macro-defects, such as internal pores and cracks under all welding conditions. Furthermore, shrinkage voids were observed in the interdendritic region near the center line of the weld metal, and the volume fraction of these voids decreased as the welding velocity increased. 

**Figure 3:**
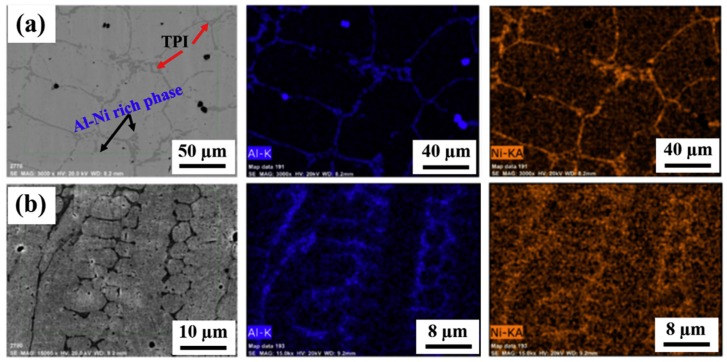
Comparison of the microstructure of the (**a**) BM and (**b**) WZ of Al_0.5_CoCrFeNi HEA. Inset of EDX mapping of Al and Ni of both regions. Adapted from [27].

Sokkalingam et al. [27] welded an Al_0.5_CoCrFeNi HEA with plate thickness of 2.5 mm while using an optic fiber laser source with a beam power and transverse speed of 1.5 kW and 600 mm min^−1^, respectively. Figure 3 shows that the laser weld resulted in a microstructure of the welding zone (WZ) with a lower degree of Al-Ni segregation in comparison with the base metal zone (BM). This microstructural difference was associated with the lack of diffusion in Al-Ni phase formation at rapid solidification. 

**Figure 4:**
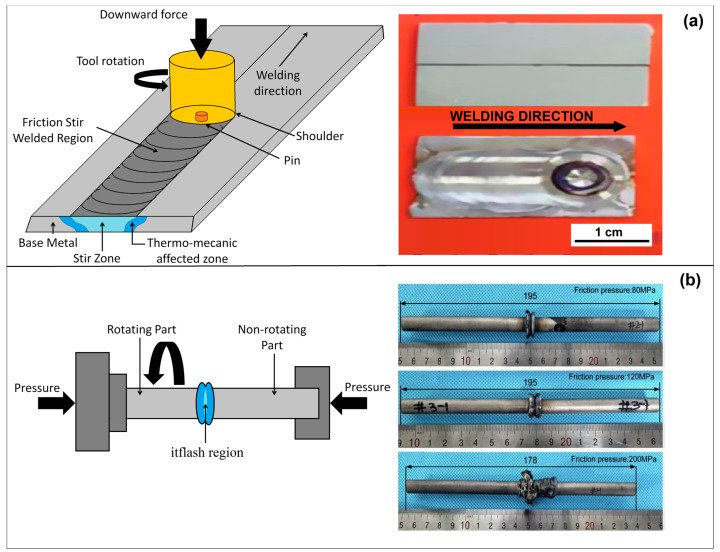
Schematic illustration and produced specimens of (**a**) friction stir (FSW) and (**b**) Rotary Friction Welding (RFW). Adapted from [29,30,31].

Regarding solid-state welding techniques for HEAs, Zhu et al. [29,30] discussed the possibility of using friction-stir welding (FSW) as welding technique to obtain sound joints in HEAs. Indeed, the authors were able to weld a Co_16_Cr_28_Fe_28_Ni_28_ and CoCrFeNiAl_0.3_ high entropy alloys while using this technique. Figure 4a shows how this process runs, the parameters, and the microstructural zones generated through this technique. The welding process was conducted using a load-controlled FSW machine with the WC-Co based welding tool. The welding speeds were 30 mm min^−1^ and 50 mm min^−1^, while the rotation rate and load force were kept constant at 400 rpm and 1500 kg, respectively. The diameter of the shoulder and pin are 12 mm and 4 mm, respectively, with a pin length of 1.8 mm. The authors reported that W-rich particles were detected within the stir zone, which was associated with the friction between the WC-Co based rotating tool and the material, although, in both cases, the weld was considered to be successful. Li et al [31] studied a variant of the Friction-Stir welding by a rotary friction welder (HSMZ-20, Harbin Welding Institute, Harbin, China) to weld an AlCoCrFeNi_2.1_ alloy. Such a welding technique is a solid-state welding process with some interesting advantages, such as: high welding productivity, low heat input, excellent welding quality, and, unlike FSW, no contamination was reported. Figure 4b illustrates the Rotary Friction Welding (RFW) process, as well as the samples that were produced by Li et al in that work. As for the parameters, the rotation speed was kept constant at 1500 rpm, while the friction pressure varies from 80 to 200 MPa. The authors showed that four different zones could be observed in the microstructure of these materials: base metal (BM), heat-affected zone (HAZ), thermal-mechanically affected zone (TMAZ), and dynamic recrystallization zone (DRZ). In addition, it was disclosed that an increase of the pressure up to 200 MPa notably enhanced the quality of the weld in a way that the tensile test for that condition resulted in the fracture being propagated in the BM zone, unlike the other conditions in which the specimens fractured in the welding zone (WZ).

**Figure 5:**
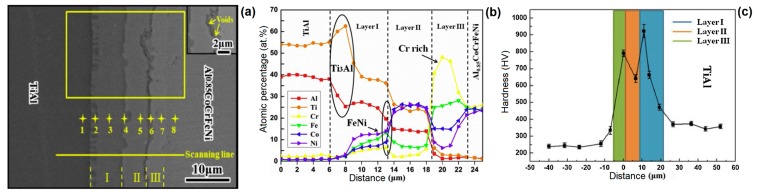
Typical insterfacial microstructure of a dissimilar diffusion bonding (**a**), compositional elemental distribution through the graded microstructura (**b**), and (**c**) measured Vickers hardness in the different layers that formed the welded joint. Adapted from [32].

Another interesting welding technique that was recently reported for HEAs is the diffusion bonding [32,33]. As one should expect, in this technique the metallurgical bonding of the alloys depend on the diffusion of the elements from one metallic block to the other. Therefore, two metallic blocks are placed in a vacuum chamber, under a certain pressure, temperature (between 0.6 and 0.8 of the melting temperature), and during a determinate time. The applied parameters depend on the materials that have been welded in this diffusion-base welding method. Lei et al. [32] investigated the dissimilar joint of a single phase face center cubic Al_0.85_CoCrFeNi alloy and a TiAl intermetallic while using direct diffusion bonding under vacuum. For this work, temperatures in the range from 750–1050 °C, holding time of 30–120 min. and constant pressure of 30 MPa, were used to evaluate the weldability of this dissimilar joint. Figure 5a shows the typical interfacial microstructure that was observed for the TiAl/Al_0.85_CoCrFeNi. One should notice that the emergence of such graded microstructure is directly related to the diffusion velocity of each element of the HEA, as well as the TiAl. The authors suggest four stages for the diffusional bonding of the HEA/TiAl joint. In the first stage, there was physical contact of the base materials with a low degree of atomic diffusion and no reaction layer formed. In the second stage, a large number of Ni and Co atoms diffused into the TiAl. Consequently, it is observed that the formation of α_2_-phase and the solid solution strengthened γ-TiAl. In the third stage, it is noticed the formation of Ti(Ni, Co)_2_Al and Cr(Fe, Ni)ss layer. At this stage, the diffusional layers are formed and a reliable metallurgical bond can be observed. Finally in the fourth stage the growth of diffusion layers takes place. This late grow could be associated with the sluggish diffusion character of FCC-structured AlCoCrFeNi HEA. Figure 5b presents the compositional elemental distribution over the graded microstructure marked with yellow points in Figure 5a. The Figure 5b helps to understand the microstructural evolution of the joint and with the appearance of intermetallic phases. Such intermetallics as Cr- rich, Ti_3_Al, and FeNi phases were held responsible for increasing the hardness in each region, as shown in Figure 5c.

## 3. Properties of Welded HEAs

The welding process of HEAs presents direct impact in several properties of such materials. In fact, many of these properties are measured to assess whether the quality of the welding was, indeed, satisfactory. Microstructure might present grain size modification, secondary phases precipitation, segregation, and lattice distortions. Therefore, corrosion, fatigue, and creep service conditions are significantly modified. In addition, mechanical properties, such as tensile strength, ductility, and hardness, are also important parameters to appraise the welding.

**Figure 6:**
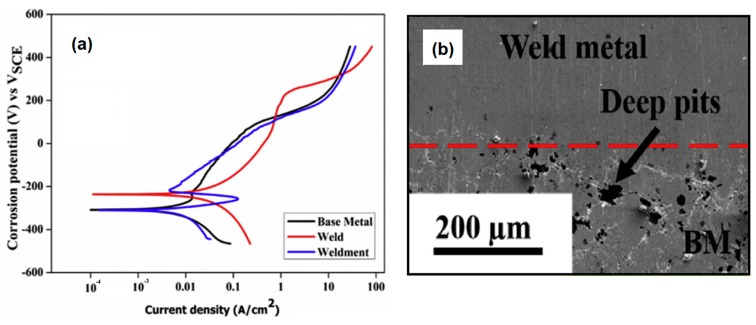
(**a**) Corrosion behavior and (**b**) deep pits near the interface between WZ and BM of Al_0.5_CoCrFeNi HEA. Adapted from [27].

Al_0.5_CoCrFeNi alloy was reported to present better corrosion resistance than 304 stainless steel, but with an increased strength [34]. Sokkalingam et al. [27] studied the corrosion resistance of that welded alloy to verify whether the welding process could deteriorate it. It was observed that the WZ exhibited higher corrosion current density (2.83 × 10^−5^ mA/cm^2^) than the BM (8.63 × 10^−6^ mA/cm^2^), which showed a higher corrosion rate. The welded joint BM+WZ resulted in the WZ acting like cathode and BM acting as anode, which could result in the secondary phases and particles in the BM been corroded first, as the weldment is exposed to a corrosive environment. Furthermore, deep pit corrosion was observed in the interface between the WZ and the BM zone. This phenomenon was associated with the dissolution of Al-rich particles at the BM zone near the interface. Figure 6a shows the corrosion behavior of such welded joint, while, in Figure 6b, one might observe the deep pit corrosion that occurs near the interface WZ+BM.

**Figure 7:**
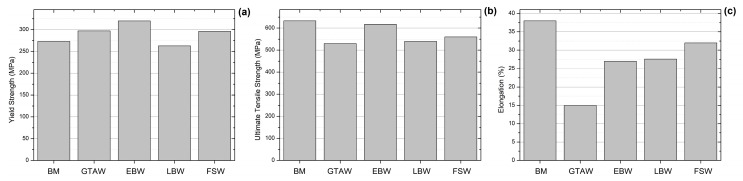
Comparison of mechanical properties of CoCrFeMnNi HEAs welded by different techniques (**a**) yield strength, (**b**) ultimate tensile strength, and (**c**) elongation.

In another study regarding the properties of HEAs welded joints, Wu et al. [24] compared the microstructure and mechanical properties of the CoCrFeMnNi alloy welded by GTAW and electron beam welding (EBW). In spite of the low process velocity for the EBW process, 38 mm min^−1^, which was compared to the GTAW process, 25.4 mm min^−1^, a remarkable difference in the microstructure could be observed in comparison to each case. The GTAW welding zone is at least 2.5 times wider than the EBW welding zone, 3.3 and 1.3 mm respectively which is, obviously, associated with the heat input in each technique. Moreover, both of the welds exhibit yield columnar grains that grows towards the maximum temperature gradient following the solid-liquid interface direction. The mechanical properties of tensile strength and ductility were analyzed, Figure 7a–c. For both GTAW and EBW conditions, the yield strain was increased when compared to the base metal, but only the EBW weld was able to keep similar ultimate tensile strength properties. For both cases, the ductility was decreased, 15% for GTAW, 27% for EBW against 38% for the BM. Furthermore, a compositional mapping of the welding produced revealed a depletion of Mn in the weld zone for the EBW technique, where the atomic percentages in the range from 13–18 at% of Mn were reported. This depletion was associated with the evaporation of Mn due to the high power density of the EBW welding process. Indeed, this should be expected, since Mn is the element with the lowest melting temperature and highest evaporation pressure in the Cantor alloy. On the other hand, the energy input for the GTAW technique did not impact the local composition of the Cantor alloy and the atomic percentage of all elements was kept around 20 at%. 

Jo et al. [28] also undertook a comparative study on the properties of CrMnFeCoNi HEA welded by FSW and LBW. It was not observed macroscopic defects for both FSW and LBW HEA. The strength and ductility of the welded specimens were comparable with that of the BM. The FSW specimen had relatively higher yield strength (296 MPa) when compared with that of the BM (272 MPa). The loss in ductility in the FSW specimen (9%) was less than that in the LBW specimen (16%) when compared with the BM, was associated with the grain refinement due to dynamic recrystallization by the FSW process. Similarly to what Wu et al. [24] reported for EBW, the LBW technique also addressed a high energy input, which leads to a fluctuation in the composition of the FZ with the emergence of Mn-rich and Fe-rich phases. The tensile fracture tended to occur in the BM away from the weld center in the FSW specimen, while, in the LBW specimen, fracture occurred in the FZ. The fracture surface in both FSW and LBW specimens showed the features of dimpled rupture, which is typical of ductile fracture. Figure 7a–c compare the mechanical properties that were reported for the Cantor alloy joint by these different welding techniques.

**Figure 8:**
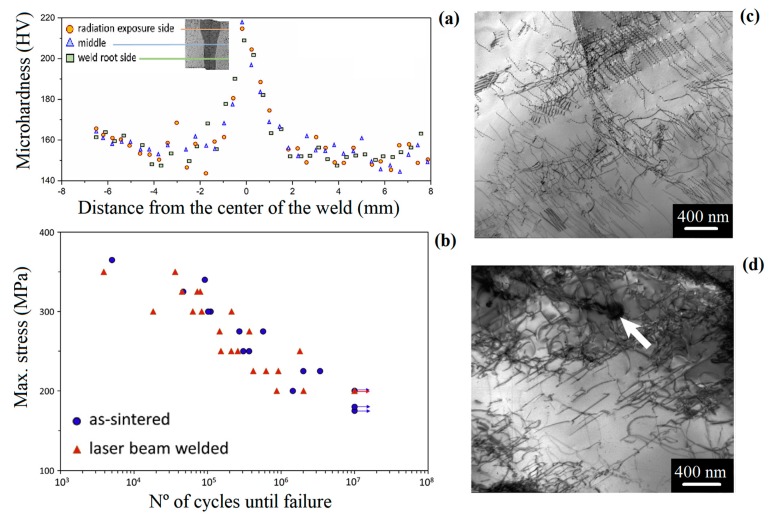
(**a**) Hardness distribution along different regions of CoCrFeMnNi HEA, (**b**) fatigue behavior of the this alloy as-sintered and LBW, TEM microstructure of the alloy (**c**) as-sintered, and (**d**) laser beam welding (LBW) after 10^7^ cycles. Adapted from [35].

Kashaev et al. [35] used LBW with a laser power of 2kW, 300μm of core diameter, 300 mm of focal length, focus position of 0.0 mm, and welding velocity in range of 3–6 m min^−1^ to butt joints CoCrFeMnNi alloys. It was observed that the welding process resulted in the precipitation of M_7_C_3_ carbides along the fcc matrix. The precipitation of this secondary phase enhanced the hardness from 150 to 205 HV, in the BM and WZ, respectively. The authors also evaluated the influence of such a welding technique in the fatigue behavior of the alloy. No significant difference was observed between the investigated conditions. Moreover an endurance limit of 200 MPa was determined for both conditions. Due to the higher strength of the weld, the failure occurs in the BM, and any possible stress concentrators in the weld as well at the WZ/HAZ or HAZ/BM boundaries do not play any significant role. Figure 8a–d present the results of that investigation. Figure 8a shows the hardness distribution of along the material. Figure 8b the behavior of the material is showed under fatigue cycles. It is important to notice that, for cycles above 10^7^, both of the conditions reached to a plateau which was associated with the endurance limit of the material. Finally, Figure 8c,d show the microstructure of the materials as-sintered (BM) and the LBW material after 10^7^ cycles, respectively. One should observe a high density of dislocation in both case, but, in Figure 8d, it is arrowed the presence of the M_7_C_3_ carbides that seem to lock the dislocations around it.

**Figure 9:**
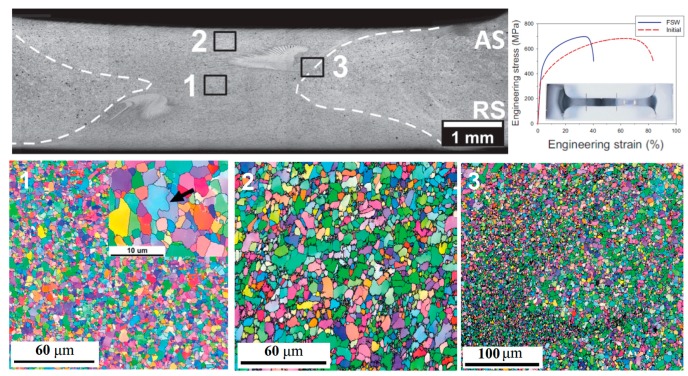
Grain size in three different regions of CoCrFeMnNi alloy welded by FSW and mechanical properties. Adapted from [36].

In some cases, even an enhancement of the properties was obtained in WZ in comparison with the BM. Shaysultanov et al. [36] used FSW to butt joint 2 mm thickness of a modified CoCrFeMnNi alloy. Along with the main elements, 0.9 at% of C was added to the alloy. This resulted in a microstructure of the HEA alloy that consisted of face centered cubic matrix and fine Cr- rich M_23_C_6_ carbides. The use of FSW for a butt-jointing of carbon-doped CoCrFeNiMn HEA specimens permitted the formation of a sound weld without any cracks or pores. Moreover, a moderate microstructure refinement was observed as the grain size in the BMl was measured to be 9.2 μm, while being 4.6 μm for the WZ. The grain refinement was claimed to be one of the advantages of the FSW and similar results were reported for the Co_16_Cr_28_Fe_28_Ni_28_ [29], CoCrFeNiAl_0.3_ [30] and CoCrFeNiMn [28]. Furthermore, this microstructural change leads to a notable enhancement of mechanical properties, such as yield strength and ultimate tensile strength. By contrast, the ductility was impaired. However, it is also important to notice that the failure occurred in the BM and not in the WZ. Figure 9 shows the grain size in three different regions along the welding joint and also display the tensile strength of both the BM and WZ.

Table 1 summarizes the main parameters, the range, and references of the alloys that were welded by each technique discussed in this short-review.

## 4. Challenges and Future Perspective

**Figure 10:**
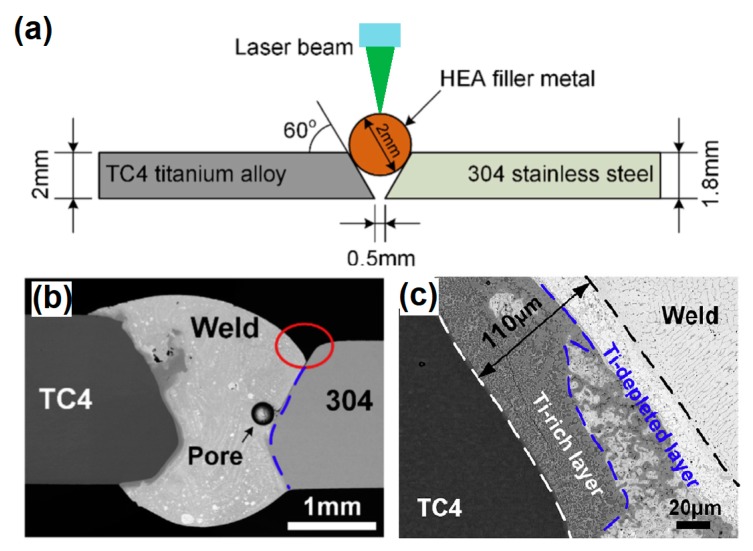
(**a**) Schematic illustration of dissimilar welding between TC4 Ti alloy and 304 stainless steel using a HEA as filler metal, (**b**) welding macroscopic aspect, and (**c**) layer formed between the WZ and the TC4 titanium. Adapted from [39].

In an innovative work, Hao et al. [39] suggested the possibility of using a (CoCrFeNi)_100-x_Cu_x_ HEA as a filler metal in a hybrid structure between 304 stainless steel and TC4 titanium alloy, as in Figure 10a. Figure 10b shows that the weld reinforcement presented some undercut defects, pores, and slag inclusion. No obvious interface could be seen between the WZ and 304 stainless steel, which suggested that reliable metallurgical bonding was achieved for these materials. However, a thin transition layer was formed between TC4 titanium alloy and the WZ. This transition layer could be divided into two different regions, a Ti-depleted and a Ti-rich layer, as shown in Figure 10c. All of the joints failed through the Ti/Cu transition zone, exhibiting a brittle nature with typical cleavage fracture characteristics. In spite of the unsuccessful attempt of joint this hybrid structure, such an approach with careful evaluation of the microstructural evolution of the BM/WZ interface as well as compositional matching between the materials to be welded and the filler metal could represent an important strategy for hybrid structure welding. 

**Figure 11:**
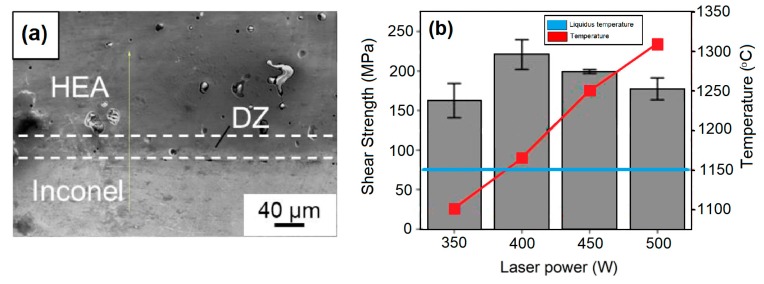
Brazing dissimilar welding between HEA and Inconel 718. (**a**) Metallurgical bond and (**b**) relationship between shear strength and brazing temperature. Adapted from [41].

The use of brazing welding has also been reported along with HEAs [40,41]. Lin et al. [40] investigated the dissimilar infrared brazing of CoCrFeMnNi equiatomic high entropy alloy and 316 stainless steel. In that study, two nickel-based fillers (BNi-2 and MBF601) were investigated as candidates for producing the CoCrFeMnNi / 316 SS joint. As expected, microstructural evolution through the joint tends to occurs and P-rich compounds were observed precipitated in the grain boundaries of the CoCrFeMnNi base metal as well as 316 SS substrate. The highest shear strength was obtained for CoCrFeMnNi / BNi-2 / 316 SS joint, 374 MPa when brazed at 1020 °C for 600 s, against 324 MPa that was obtained for the CoCrFeMnNi/MBF601/316 SS brazed at 1080 °C for 600 s. Using a different approach, Bridges et al. [41] studied the parameters and effects of using a NiMnFeCoCu HEA as a filler metal for laser brazing Inconel® 718 nickel superalloy. The authors were able to achieve a reliable metallurgical bond, Figure 11a. Moreover, it was observed that, if the brazing temperature is way above the Liquidus temperature, the shear strength of the brazing decreased, as shown in Figure 11b.

**Figure 12:**
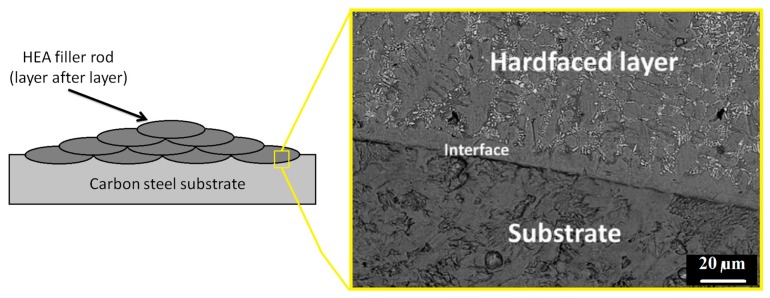
Schematic illustration of the use of HEA welded layer after layer in a carbon steel substrate and inset of the formed interface. Adapted from [37].

Abed et al. [37] used the GTAW process to add a hardfacing HEA layer into a carbon steel substrate. This could be understood as another interesting strategy for the welding of HEAs into different substrate, as the HEA filler rod was added layer after layer, as in Figure 12a. In this investigation a Fe_49_Cr_18_Mo_7_B_16_C_4_Nb_6_ was the chosen HEA filler material. The produced microstructure consisted of α-Fe matrix with Mo_2_FeB_2_ and NbC precipitated particles that substantially increased the hardness and wear resistance of the material. It was observed high-quality multi-layer deposits free of cracking with an excellent metallurgical bonding to the carbon steel base metal, as in Figure 12b.

Many interesting techniques, properties, and possibilities have been discussed regarding the welding in HEAs in the present work. Yet, one should be aware that most of the reported papers are based on the application of the Cantor alloy, its variants, or its modification. The CoCrFeMnNi HEA is, by far, the most investigated among all possible alloys due to both its properties at room and cryogenic temperature, compromise between strength and toughness, as well as the synergy of their main elements [42]. Nevertheless, this is just one possibility in a limitless compositional hyper-space. Miracle and Senkov [43] classified the HEAs in seven possible families and suggested that over 500,000 HEAs are possible to be produced if only equiatomic configurations with five main elements be considered. Hence, it is clear that, so far, only a minimum number of the potential application of HEAs is reported and discussed. This is particularly true regarding the welding of such materials, where only the “easiest” cases have been investigated so far. 

The welding of HEAs, in which the main elements present significant differences in the melting temperature could be considered as a big challenge. Chen et al. [44] suggested that the evaporation of elements with low melting points lead to a difficult proper control of the chemical composition of the produced alloy. The aforementioned work from Wu et al. [24] proves that hypothesis. In fact, in their work was revealed the depletion of Mn in the WZ due to the high energy input through the EBW welding and the lowest melting temperature of Mn when compared to the other main elements in the Cantor alloy. Moreover, Stepanov et al. [45] was able to produce an AlNbTiV high entropy refractory alloy with over 1 GPa of compression strength and density of approximately 5.6 g/cm^3^. Despite these remarkable properties, one should be wondering how to weld such material, once the melting temperature for Nb is 247 °C and the vaporization temperature for Al is 2470 °C. Adapting solid-state welding techniques, such as FSW or RFW, could come up with possible solutions for this case. New processing techniques, such as metal additive manufacturing that are emerging rapidly could be helpful in the manufacturing of HEAs with controllable microstructure and enhanced properties, as well as high complex geometry components and high freedom of design [46]. In this layer-wise fabrication process, the metallic materials are bonded together by sintering or melting using high energy source, such as high power laser, electron beam, or plasma arc. Joseph et al. [47] faced some difficult obtain desired microstructure of an Al_0.6_CoCrFeNi that is produced by direct laser deposition (laser power of 800 W, beam focus diameter of 4mm, and velocity of 800 mm min^−1^). The reason was associated with the higher cooling rate and much larger thermal gradient than traditional methods, such as arc melting. It is interesting to notice that the parameters are in the same magnitude of LBW, as showed in Table 1. Therefore, this process could be seen as a localized, layer-by-layer welding technique. In fact, most of challenges faced on the welding of HEAs are also observed for additive manufacturing of these alloys. Ocelik et al. [48] focused their study on the effects of laser processing parameters in the manufacturing of an AlCoCrFeNi HEA. Deviation from the original chemical composition, as the concentration of an element would be higher the lower its melting point, and porosity were some of the commonly reported defects. Thus, understanding and optimizing the parameters could be beneficial for both additive manufacturing and LBW in HEAs.

Finally, other important points to be looked into include the geometry of the welded specimens, as well as heat treatment and protection against possible contamination during the welding. In fact, most of the papers about HEAs welding discussed in this short-review limited their investigation to the butt-joint configuration, which is the simplest possible one. Complex configurations, closer to service conditions, such as corner, edge, and T- joints, and how heat input and amount of energy would impact in the quality of the welded joint should be further studied. The investigation on different heat treatment conditions, with or without pre- or post-weld heating associated with the welding technique, should be carried out in order to verify the effects on the mechanical properties and microstructure for each condition of HEAs welding.

## 5. Conclusions

The present short-review discussed the recent development in the welding of high entropy alloys (HEAs) and how the different possible welding techniques impact in the microstructure, mechanical properties, and service conditions, such as fatigue and corrosion behavior in this novel class of materials.

- Several techniques were reported to produce reliable metallurgical bonding in HEAs. Liquid-state welding, such as GTAW, LBW, and EBW, as well as solid-state welding, such as FSW, RSW, and diffusion bonding were discussed.
- Grain refinement, dynamic recrystallization, hardness enhancement, secondary phase precipitation, ductility decrease, and strengthening of the material were some of the detail-discussed phenomenon associated with the welding of HEAs. Moreover, the CoCrFeMnNi, which is one of most study HEAs, was also compared in terms of different welding techniques and parameter as well as mechanical properties and microstructural evolution.
- For welding techniques with high energy input, such as LBW and EBW, the loss of elements with low melting and evaporation points, such as Mn and Al, was presented as a challenge for controlling the chemical composition of the desired alloy.
- Different approaches were presented for the use of HEAs as filler materials for hybrid structure, as brazing welding dissimilar joints and layer-by-layer welding in comparison to additive manufacturing.

Finally, this specific field of welding still walks in baby steps, as the HEAs presents limitless possibilities of properties and perspectives for near future applications.

## Figures and Tables

**Table 1:** Welding parameters and techniques reported for high entropy alloys (HEAs).

	Welding Technique	Welding Parameters																References
Liquid-state Welding	GTAW	Voltage (V)			Current (A)					Velocity (mm min^−1^)					Gas Shield (L min^−1^)			[23,24,37,38]
		8.4–16			40–75					25.4 - 80					0 - 16			
	LBW	Power (kW)			Focus (mm)					Velocity (mm min^−1^)					Gas Shield (L min^−1^)			[25,26,27,28,35,39]
		1.5–3.5			0–2					600–10000					0–40			
	EBW	Voltage (kV)					Current (mA)						Velocity (mm min^−1^)					[22,24]
		125					2.2–5.0						38–570					
Solid-state Welding	FSW	Rotation (rpm)	Shoulder Diameter (mm)			Pin Length (mm)			Pin Diameter (mm)		Force (kgf)			Tilt Angle (°)			Velocity (mm min^−1^)	[28,29,30,36]
		400 -1000	12–12.5			1.5–1.85			4–5.76		1130–1500			2–3			30–150	
	RSW	Friction Pressure (MPa)		Friction Time (s)				Forging Pressure (MPa)				Forging Time (s)				Rotation Speed (rpm)		[31]
		80–200		3				120–400				15				1500		
	Diffusion Bonding	Temperature (°C)			Time (min)					Pressure (MPa)					Dissimilar Welding			[32,33]
		750–1050			30–120					15–30					TiAl alloy			
